# Nonsmooth analysis of three-dimensional slipping and rolling in the presence of dry friction

**DOI:** 10.1007/s11071-019-04913-x

**Published:** 2019-04-04

**Authors:** Mate Antali, Gabor Stepan

**Affiliations:** Department of Applied Mechanics, Budapest University of Technology and Economics, Budapest, 1521 Hungary

**Keywords:** Nonsmooth dynamics, Filippov systems, Coulomb friction, Slipping

## Abstract

In this paper, the nonsmooth dynamics of two contacting rigid bodies is analysed in the presence of dry friction. In three dimensions, slipping can occur in continuously many directions. Then, the Coulomb friction model leads to a system of differential equations, which has a codimension-2 discontinuity set in the phase space. The new theory of extended Filippov systems is applied to analyse the dynamics of a rigid body moving on a fixed rigid plane to explore the possible transitions between the slipping and rolling behaviour. The paper focuses on finding the so-called limit directions of the slipping equations at the discontinuity. This leads to a complete qualitative description of the possible scenarios of the dynamics in the vicinity of the discontinuity. It is shown that the new approach consistently extends the information provided from the static friction force of the rolling behaviour. The methods are demonstrated on an application example.

## Introduction

If dry friction is assumed between the surfaces of rigid bodies, the dynamical model of the bodies leads to discontinuous behaviour. By considering the simple Coulomb model in the two-dimensional (2D) contact problems, the friction force changes sign at zero relative velocity of the surfaces. The situation is similar but more complicated in the three-dimensional (3D) contact dynamics. Then, for infinitesimally small relative velocities, the Coulomb friction model provides continuously many directions of the friction force with a constant finite amplitude.

The direct substitution of the discontinuous friction models into the dynamical equations leads to discontinuous systems of differential equations. In the 2D case, the Coulomb friction leads to *Filippov systems* (for an overview and examples, see [7]). Considering the friction as a set-valued force law leads to *differential inclusions*, which is a completely different point of view of modelling (see [13] for an overview). A further approach can be found in [6, 16, 17].

The generalization of the Filippov systems to codimension-2 discontinuity sets in the phase space leads to the concept of *extended Filippov systems* (see [1] and [4]). This type of differential equation can be used for modelling and analysis of 3D mechanical systems with dry friction, which was demonstrated in specific mechanical examples in [4]. The early results about two general contacting bodies have been presented by the authors in [3].

In this paper, we analyse the dynamics of a single rigid body in contact with a fixed rigid plane. During the motion of the body, rolling or slipping can occur, and the slipping case reveals to be described by an extended Filippov system. We focus on the transitions between the slipping and rolling dynamics when applying the theory of extended Filippov systems. The so-called *limit directions* can be determined, which are strongly connected to the possible slipping–rolling transitions. One of our main motivations is to provide a deeper understanding of the qualitative dynamics in the neighbourhood of the discontinuity. But the results makes the possibility for new numerical methods for simulating these mechanical systems, as well.

The paper is organized as follows: In Sect. 2, the dynamic equations of the moving body are derived by appropriate choice of the state variables for the subsequent analysis. In Sect. 3, the basic concepts and definitions of extended Filippov systems are presented. The main part of the paper is Sect. 4, where the theory of extended Filippov systems is applied to the dynamics of the moving body. From the analysis, we get four typical cases of the limit directions. In Sect. 5, the mechanical consequence of the four cases is explained, and the relation with the static friction force is presented. In Sect. 6, the results are demonstrated on a mechanical example. In Sect. 7, an overview can be found about the possible extension of the results to more complicated contact models.

This paper is a significantly extended version of the conference paper [3]. The content of Sects. 2–5 has been rearranged and improved, and most importantly, the former conjectures have been developed into a series of proved statements about the possible slipping–rolling transitions, as can be found in the current paper. Sections 6 and 7 are completely new.

## Dynamics of a rigid body on a flat surface

**Fig. 1:**
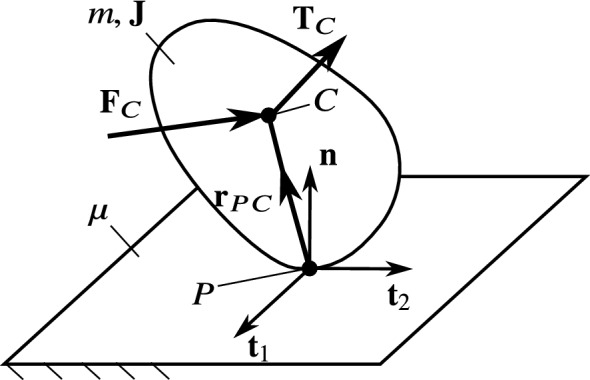
Sketch of the analysed mechanical scenario. A rigid body is moving in contact with a fixed plane. In this paper, the transitions between slipping and rolling are investigated

We consider a rigid body moving in contact with a fixed rigid plane (see Fig. 1). It is assumed that the at any moment, the body and the plane are touching each other in a single contact point, denoted by *P*. The centre of the gravity of the body is denoted by *C*. The notation of the important quantities are summarized in Table 1.

**Table 1:** Important notation of the mechanical system

Notation	Quantity
*C*	Centre of gravity of the rigid body
*P*	Contact point
$\mathbf {r}_{PC}$	Position vector between *P* and *C*
*m*	Mass of the body
$\mathbf {J}$	Mass moment of inertia of the body
$\mu$	Friction coefficient between the surfaces
$\mathbf {F}_C,\mathbf {T}_C$	Resultant force and torque of external forces computed at *C*
$\mathbf {n}$	Normal unit vector at *P*
$\mathbf {t}_1,\mathbf {t}_2$	Tangential unit vectors at *P*
$N\mathbf {n}$	Normal force at *P*
$\mathbf {F}_f$	Friction force at *P*
$\mathbf {F}_P$	Total contact force at *P*
$\rho _n$	Reciprocal of the normal curvature of the body in the direction of motion
$\mathbf {v}_C,\mathbf {v}_P$	Velocities of *C* and *P*
$u_1,u_2$	Components of the velocity $\mathbf {v}_P$ (slipping)
${{\varvec{\omega }}}$	Angular velocity of the body
$\omega _1,\omega _2,\omega _3$	Components of ${{\varvec{\omega }}}$
*q*	Vector of generalized coordinates
*s*	Vector of quasi-velocities

### Kinematics

Let $\mathbf {v}_C$ and $\mathbf {v}_P$ denote the velocities of the points *C* and *P*, respectively, and ${{\varvec{\omega }}}$ denotes the angular velocity of the body. The relation between these quantities of the rigid body is given by

1 $$\begin{aligned} \mathbf {v}_C=\mathbf {v}_P+{{\varvec{\omega }}}\times \mathbf {r}_{PC}, \end{aligned}$$

where $\mathbf {r}_{PC}$ is the position vector of *C* measured from the contact point *P*.

During the motion of the body, the contact point *P* corresponds to different material points of the rigid body. We can take the time derivatives of (1) by using two different approaches: either by following the *material point of the body currently located at P*, or, following the motion of the *instantaneous geometric contact point P*.

In the first case of considering *P* as a *material point*, the differentiation of (1) gives the usual acceleration formula

2 $$\begin{aligned} \mathbf {a}_C=\mathbf {a}_P+\dot{{{\varvec{\omega }}}}\times \mathbf {r}_{PC} +{{\varvec{\omega }}}\times (\mathbf {v}_C-\mathbf {v}_P), \end{aligned}$$

where $\mathbf {a}_C$ and $\mathbf {a}_P$ denote the acceleration of the points *C* and *P*, respectively, and $\dot{{{\varvec{\omega }}}}$ denotes the angular acceleration of the body.

However, when differentiating (1) by considering *P* as the *contact point*, we get

3 $$\begin{aligned} \mathbf {a}_C=\dot{\mathbf {v}}_P+\dot{{{\varvec{\omega }}}}\times \mathbf {r}_{PC} +{{\varvec{\omega }}}\times \dot{\mathbf {r}}_{PC}. \end{aligned}$$

The time derivative $\dot{\mathbf {v}}_P$ is the rate of change of the velocity of the instantaneous contact point *P*, which is not equal to the acceleration $\mathbf {a}_P$ of the material point of *P* in (2). The time derivative of $\mathbf {r}_{PC}$ can be written as

4 $$\begin{aligned} \dot{\mathbf {r}}_{PC}=\mathbf {v}_C-(\mathbf {v}_P+\mathbf {w}_P). \end{aligned}$$

In the bracket, the two terms are the velocity $\mathbf {v}_P$ of the motion of the material point at *P* and the velocity $\mathbf {w}_P$ of the *translation of the contact point* on the surface of the body. This latter quantity depends on the rotation and the local curvature of the body, and it can be calculated by

5 $$\begin{aligned} \mathbf {w}_{P}=\rho _n\cdot \left( {{\varvec{\omega }}}\times \mathbf {n}\right) , \end{aligned}$$

where $\mathbf {n}$ is the unit normal vector of the rigid plane and $\rho _n$ denotes the reciprocal of the normal curvature of the surface of the body in the plane determined by ${{\varvec{\omega }}}\times \mathbf {n}$. In the general case, the normal curvature is determined by the second fundamental form of the surface of the body (see [12], p. 206). In the case of simple geometries, the quantities $\rho$ or $\mathbf {w}_P$ can be often found intuitively.

What is the point of writing $\mathbf {a}_C$ in the form of (3)? In the subsequent calculations, we are using the components of $\mathbf {v}_P$ as phase variables. As the Coulomb friction model is discontinuous exactly at $\mathbf {v}_P=\mathbf {0}$, this choice of variables, the discontinuity set of the resulting differential equation can be treated easily.

### Dynamics

The effect of the rigid plane on the body is modelled by a single force $\mathbf {F}_P$ acting at *P*. This force can be separated into a normal force $N\mathbf {n}$ and a friction force $\mathbf {F}_f$ in the form

6 $$\begin{aligned} \mathbf {F}_P=N\,\mathbf {n}+\mathbf {F}_f \end{aligned}$$

We assume that the normal force *N* is strictly positive and the body remains in permanent contact with the plane (the scalar product $\mathbf {v}_P\cdot \mathbf {n}$ is zero). That is, the effects of loosing contact, impact without collision, and consistency problems of the Painleve paradox (see [11, 15]) are excluded from this analysis.

All other forces and torques acting on the body are substituted by a single force $\mathbf {F}_C$ and a torque $\mathbf {T}_C$ acting at the centre of gravity *C*. Let *m* denote the mass of the body and $\mathbf {J}$ is the moment of inertia tensor with respect to the point *C*. Then, the Newton–Euler equations of the body are

7 $$\begin{aligned} \begin{aligned}&m\mathbf {a}_C=\mathbf {F}_C+\mathbf {F}_P,\\&\mathbf {J}\dot{{{\varvec{\omega }}}}+{{\varvec{\omega }}} \times \left( \mathbf {J}{{\varvec{\omega }}}\right) =\mathbf {T}_C-\mathbf {r}_{PC}\times \mathbf {F}_P. \end{aligned} \end{aligned}$$

In addition, we have to consider supplementary conditions about contact between the body and the plane, according to the rolling or slipping. In the case of *rolling*, the kinematic constraint

8 $$\begin{aligned} \mathbf {v}_P= 0 \end{aligned}$$

is satisfied. In the case of *slipping*, the friction force $\mathbf {F}_f$ is modelled by the three-dimensional Coulomb friction law,

9 $$\begin{aligned} \mathbf {F}_f=-\mu N\frac{\mathbf {v}_P}{|\mathbf {v}_P|}, \end{aligned}$$

where $\mu$ denotes the friction coefficient.

Equations (6)–(9) lead to a system of differential equations in the case of rolling or slipping. In this paper, we focus on the *slipping* equations with special attention to their behaviour close to the rolling state.

### Differential equations for the slipping case

By considering (1)–(5), the Newton–Euler equations (7) can be written into the form

10 $$\begin{aligned}&\dot{{{\varvec{\omega }}}}=\mathbf {J}^{-1}\cdot \left( -{{\varvec{\omega }}} \times \left( \mathbf {J}{{\varvec{\omega }}}\right) +\mathbf {T}_C-\mathbf {r}_{PC}\times \mathbf {F}_P\right) ,\end{aligned}$$

11 $$\begin{aligned}&\dot{\mathbf {v}}_P = \tfrac{\mathbf {F}_C+\mathbf {F}_P}{m} -\dot{{{\varvec{\omega }}}}\times \mathbf {r}_{PC}-{{\varvec{\omega }}} \times \left( {{\varvec{\omega }}}\times \left( \mathbf {r}_{PC}-\rho _n\mathbf {n}\right) \right) .\nonumber \\ \end{aligned}$$

We introduce coordinates both on the displacement and the velocity level. Thus, a set of first-order ordinary differential equations (ODEs) is obtained from (10)–(11).

In the vicinity of a chosen initial state, the position and the orientation of the rigid body by five generalized coordinates; the sixth degree of freedom is constrained by the contact between the body and the plane. Let the generalized coordinates be denoted by

12 $$\begin{aligned} q=(q_1,q_2,q_3,q_4,q_5). \end{aligned}$$

Note that along the paper, the vectors with a *three-dimensional physical meaning* are denoted by boldface symbols (e.g. $\mathbf {F}_C,\mathbf {v}_P$), but all other vectors are not distinguished from scalars in notation (e.g. *q*, *s*, *x*).

Instead of describing the velocity level by the time derivatives of (12), we choose quasi-velocities (see [14], p. 254) independently from the generalized coordinates (12) to describe the velocity state of the body. By choosing two orthogonal unit vectors $\mathbf {t}_1$ and $\mathbf {t}_2$ parallel to the rigid plane (see Fig. 1), we get an orthonormal basis $(\mathbf {t}_1,\mathbf {t}_2,\mathbf {n})$. In this coordinate system, $\mathbf {v}_P$ and ${{\varvec{\omega }}}$ can be written as

13 $$\begin{aligned} \mathbf {v}_P=u_1\mathbf {t}_1+u_2\mathbf {t}_2,&{{\varvec{\omega }}}=\omega _1\mathbf {t}_1+\omega _2\mathbf {t}_2+\omega _3\mathbf {n}, \end{aligned}$$

where the components are chosen as quasi-velocities in the form

14 $$\begin{aligned} s=(u_1,u_2,\omega _1,\omega _2,\omega _3) \end{aligned}$$

These five linearly independent variables fully describe the velocity state of the body for any state of general coordinates. That is, the time derivatives of the generalized coordinates can be written as

15 $$\begin{aligned} \dot{q}= K(q)\cdot s, \end{aligned}$$

where *K*(*q*) is a 5-by-5 matrix depending on the generalized coordinates themselves.

By taking the time derivative of (13), we get

16 $$\begin{aligned} \dot{\mathbf {v}}_P=\dot{u}_1\mathbf {t}_1+\dot{u}_2\mathbf {t}_2, \qquad \dot{{{\varvec{\omega }}}}=\dot{\omega }_1\mathbf {t}_1+\dot{\omega }_2\mathbf {t}_2+\dot{\omega }_3\mathbf {n}, \end{aligned}$$

which can be substituted into the left-hand sides of (10) and (11). On the right-hand sides of (10)–(11), all quantities can be expressed by *q*, *s*,  and *N* in the following way: The geometric quantities $\mathbf {r}_{PC}(q)$ and $\rho _n(q)$ depend on the generalized coordinates. The moment of inertia tensor $\mathbf {J}(q)$ depends on *q* as well, because of the change of the orientation of the body. With the assumption of *no explicit time dependence* in the external forces, the resultants $\mathbf {F}_C(q,s)$ and $\mathbf {T}_C(q,s)$ are expressed by *q* and *s*. In the *slipping* case, (6),(9), and (13) leads to

17 $$\begin{aligned} \mathbf {F}_P= -\mu N\tfrac{u_1}{\sqrt{u_1^2+u_2^2}}\,\mathbf {t}_1 -\mu N\tfrac{u_2}{\sqrt{u_1^2+u_2^2}}\,\mathbf {t}_2 +N\mathbf {n}. \end{aligned}$$

**Table 2:** Important notation of extended Filippov systems

Notation	Quantity
$\mathcal {D}$	Phase space of the system (subset of $\mathbb {R}^m$)
*x*	Vector of phase variables (element in $\mathcal {D}$)
*F*(*x*)	Vector field of the system
$\Sigma$	Discontinuity manifold of *F*(*x*) ($m-2$-dimensional subset of $\mathcal {D}$)
$\mathcal {T}_{x_0}\Sigma$	Tangent space of $\Sigma$ at $x_0$
$\mathcal {O}_{x_0}\Sigma$	Orthogonal space of $\Sigma$ at $x_0$
$n_1,n_2$	Orthogonal basis vectors of $\mathcal {O}_{x_0}\Sigma$ at $x_0$
$\phi$	Aangle parametrizing the directions of $\mathcal {O}_{x_0}\Sigma$ around $x_0$
$n(\phi )$	Set of unit normal vectors to $\Sigma$ at $x_0$
$F^*(\phi )(x_0)$	Limit vector field (directional limit of *F* at $x_0$ from the different directions $\phi$)
$R(\phi )$	Radial component of $F^*(\phi )$
$V(\phi )$	Circumferential component of $F^*(\phi )$

Consequently, Eqs. (15), (10), and (11) form a set of six differential-algebraic equations in the generalized coordinates (12), the quasi-velocities (14), and the normal force *N*.

Equations  (10)–(11) are linear in the derivatives of the quasi-velocities and *N*; it can be solved in the form

18 $$\begin{aligned} \dot{s}&=f_s(q,s), \end{aligned}$$

19 $$\begin{aligned} N&=f_N(q,s), \end{aligned}$$

where $f_s(q,s)$ and $f_N(q,s)$ denote the formal dependence on the variables.

Equations  (15) and (18) form a system of ten first-order ODEs for the variables (12) and (14). Due to the discontinuity of the contact force (17), the system is not defined at $u_1=u_2=0$, which corresponds to the *rolling* behaviour. For the rolling states, a different set of differential equations can be derived by excluding the slipping Coulomb law (9) but including the rolling constraint (8). To obtain a deeper insight to the switches between rolling and slipping, this paper focuses on the analysis of *slipping* system (15) and (18) in the vicinity of the discontinuity $u_1=u_2=0$.

Note that this discontinuity is located at the states where two variables ($u_1$ and $u_2$) are zero *at the same time*. For the analysis of differential equations with such discontinuity, we can use effectively the theory of *extended Filippov systems*, which is presented briefly in the next section.

## Overview of extended Filippov systems

The concept of *extended Filippov systems* was introduced by the authors in [1] and [4]. Roughly speaking, these dynamical systems are vector fields containing $m-2$-dimensional discontinuities in the *m*-dimensional phase space. We will show in Sect. 4 that the contact problem of the rigid body presented in Sect. 2 leads to an extended Filippov system.

In this section, only the most important concepts and definitions of these dynamical systems are presented, which are utilized in the subsequent analysis of the mechanical system. For a more detailed presentation of the theory of extended Filippov systems, see [1] and [4]. The notation of the important corresponding quantities can be found in Table 2.

**Fig. 2:**
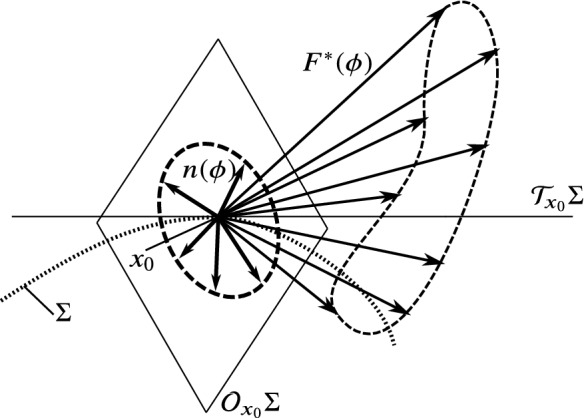
Basic concepts of extended Filippov systems. The codimension-2 discontinuity set $\Sigma$ is depicted as a curve (1D) in a 3D phase space, but it possesses more dimensions in higher dimensional systems. There are continuously many unit vectors *n* being perpendicular to the discontinuity set at any point $x_0$. The possible normal directions are parameterized by an angle $\phi$. The vector field *F* is discontinuous at $\Sigma$, and it possesses a directional limit $F^*(\phi )$ for any direction $\phi$. The set of these limits is called the limit vector field

Consider a domain $\mathcal {D}\in \mathbb {R}^m$ containing an $m-2$-dimensional smooth manifold $\Sigma \subset \mathcal {D}$. This *codimension-2* subset consists of the points where the vector field of the system is discontinuous in the sense of the following definition. At a chosen point $x_0\in \Sigma$, let us denote the tangent space by $\mathcal {T}_{x_0}\Sigma$, and its orthogonal complement by $\mathcal {O}_{x_0}\Sigma$ (see Fig. 2). By considering the usual scalar product $\left\langle .,.\right\rangle$ in $\mathbb {R}^m$, the orthogonal complement is defined by

20 $$\begin{aligned} \mathcal {O}_{x_0}\Sigma :=\left\{ v\in \mathbb {R}^m : \left\langle v,w\right\rangle =0 \quad \forall w\in \mathcal {T}_{x_0}\Sigma \right\} . \end{aligned}$$

Consequently, the direct product of the two-dimensional $\mathcal {T}_{x_0}\Sigma$ and the $m-2$-dimensional $\mathcal {O}_{x_0}\Sigma$ spans the whole vector space $\mathbb {R}^m$.

Consider a point $x_0\in \Sigma$ and choose two vectors $n_1(x_0)$ and $n_2(x_0)$ in $\mathcal {O}_{x_0}\Sigma$ depending smoothly on $x_0$ with $\left\langle n_1,n_1\right\rangle =\left\langle n_2,n_2\right\rangle =1$ and $\left\langle n_1,n_2\right\rangle$=0. In other words, $n_1(x_0)$ and $n_2(x_0)$ generate an orthonormal basis of $\mathcal {O}_{x_0}\Sigma$. Then, let us define

21 $$\begin{aligned} n(\phi )(x_0):=\cos \phi \cdot n_1(x_0)+\sin \phi \cdot n_2(x_0), \end{aligned}$$

in which function $n(\phi )$ maps the interval $[0,2\pi )$ onto the set unit vectors of $\mathcal {O}_{x_0}\Sigma$. The parameter $\phi \in [0,2\pi )$ can be imagined as an angle corresponding to a direction which is orthogonal to $\Sigma$ at $x_0$ (see Fig. 2).

### Definition 1

Consider the vector field

22 $$\begin{aligned} \dot{x}=F(x),&\quad x\in \mathcal {D}\subset \mathbb {R}^m,&F:\mathcal {D}{\setminus }\Sigma \rightarrow \mathbb {R}^m, \end{aligned}$$

with the $m-2$-dimensional smooth manifold $\Sigma$. The system (22) is called an ***extended Filippov system*** if the following properties are satisfied:

- The vector field *F* is smooth on $\mathcal {D}{\setminus }\Sigma$.
- The limit
  23 $$\begin{aligned} F^*(\phi )(x_0):=\lim _{\epsilon \rightarrow 0^+} F\left( x_0+\epsilon n(\phi )(x_0)\right) \end{aligned}$$
  exists for all $x_0\in \Sigma$ and for all $\phi \in [0,2\pi )$.
- For all $x_0$, there exist $\phi _1,\phi _2\in [0,2\pi )$ for that $F^*(\phi _1)\ne F^*(\phi _2)$.

In the sense of Definition 1, $\Sigma$ is called a ***codimension-2 discontinuity manifold of***${{\varvec{F}}}$. At a chosen point $x_0\in \Sigma$, the function $F^*(\phi )$ is called the ***limit vector field*** of *F* (see Fig. 2), which contains the directional limits of *F* from the different directions parameterized by $\phi$.

The three conditions of Definition 1 formally express that there is no discontinuity outside $\Sigma$ (see (a)), there is indeed discontinuity at any point of $\Sigma$ (see (c)), and the directional limit does not diverge from any direction (see (b)).

By projecting the limit vector field $F^*(\phi )$ on $\mathcal {O}_{x_0}\Sigma$, we get the components

24 $$\begin{aligned} F^*_1(\phi ):=\left\langle F^*(\phi ),n_1\right\rangle ,&F^*_2(\phi ):=\left\langle F^*(\phi ),n_2\right\rangle . \end{aligned}$$

For the subsequent analysis, it is useful to write up the components of $F^*(\phi )$ considering the component being parallel and perpendicular to the corresponding normal vector $n(\phi )$. Let us define

25 $$\begin{aligned} R(\phi )&:=\left\langle F^*(\phi ),n(\phi )\right\rangle , \end{aligned}$$

26 $$\begin{aligned} V(\phi )&:=\left\langle F^*(\phi ),n(\phi +\pi /2)\right\rangle . \end{aligned}$$

**Fig. 3:**
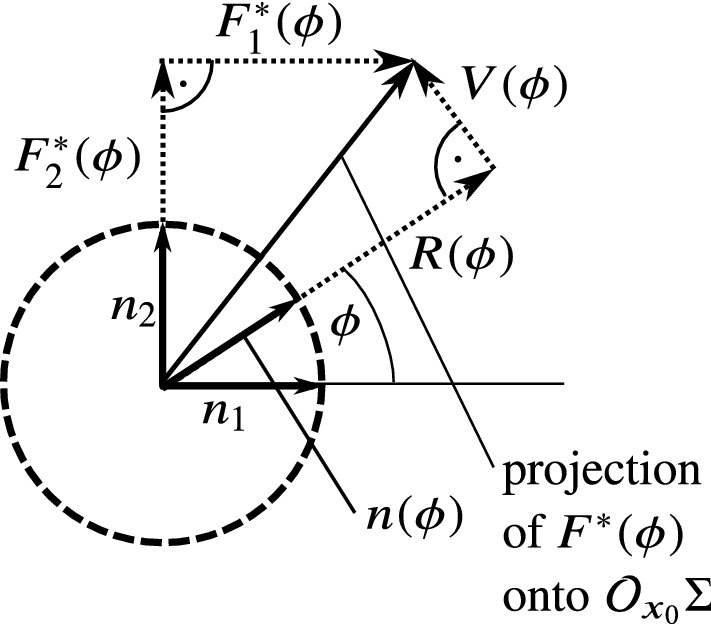
Quantities of the vector field defined in the orthogonal space $\mathcal {O}_{x_0}\Sigma$ of $x_0\in \Sigma$. For a given $\phi \in [0,2\pi )$, the value $F^*(\phi )$ is projected into $\mathcal {O}_{x_0}\Sigma$ of $x_0\in \Sigma$. This vector can be separated to the components $R(\phi )$ and $V(\phi )$ by using the direction of the corresponding normal vector $n(\phi )$. The function *R* gives the *radial* behaviour of the vector field around the discontinuity, and *V* expresses the *circumferential* behaviour, which is the key of finding the limit directions

The function $R(\phi )$ contains the behaviour of vector field in the radially *inward or outward* direction. The function $V(\phi )$ gives the circumferential, *rotating behaviour* of the vector field around the discontinuity set (see Fig. 3).

Let us now define the concept of limit directions, which are strongly connected to the behaviour of the trajectories at the discontinuity.

### Definition 2

Consider an extended Filippov system $\dot{x}=F(x)$ and a point $x_0\in \Sigma$ of the discontinuity manifold. The roots of the equation $V(\phi )=0$ are called the ***limit directions*** of $x_0$ with respect to *F*.

### Definition 3

A limit direction $\phi _1$ with $V(\phi _1)=0$ is called ***attracting*** if $R(\phi _1)<0,$ and it is called ***repelling*** if $R(\phi _1)>0$.

It can be proved (see [4]) that if $x_0\in \Sigma$ possesses at least one limit direction, then all trajectories tending to $x_0$ (either forward or backward time) approach $x_0$ along the limit directions.

In this sense, the limit directions are somewhat analogous to the eigenvectors of equilibrium points of smooth systems, but there are fundamental differences. Firstly, an eigenvector of a saddle or node is bi-directional (corresponding to a line), while a limit direction is uni-directional (corresponding to a half-line). Secondly, the eigenvectors of equilibria correspond to infinite-time (exponential) convergence of the solutions in forward or backward time, while trajectories reach $x_0$ in *finite* time in forward (attracting) or backward (repelling) direction of time.

By continuing the analogy with the equilibria, we can separate the node-like (*sliding*) and saddle-like (*crossing*) behaviour.

### Definition 4

Consider a point $x_0\in \Sigma$ which possesses at least one limit direction. If all the limit directions are either attracting or repelling, then we say that $x_0$ is located in the ***sliding region*** of $\Sigma$. If there is at least one attracting and one repelling limit direction, then we say that $x_0$ is located in the ***crossing region*** of $\Sigma$.

The terminology of *crossing* and *sliding* was introduced in [1] and [4] by generalizing of the crossing and sliding region of classical Filippov systems with codimension-1 discontinuities (see [7]). In the crossing case, there is at least one incoming and one leaving half-trajectory at $x_0$, which can be concatenated to a trajectory crossing through $\Sigma$ at $x_0$. In the sliding case, there are either only incoming or only leaving trajectories and the dynamics of *F* gets stuck into $\Sigma$ in forward or backward time, respectively. Then, the so-called ***sliding dynamics*** generated inside the discontinuity manifold $\Sigma$. For the derivations and a more detailed explanation, see [4].

The introduction of the extended Filippov systems was originally motivated by 3D contact problems of rigid bodies. In these mechanical problems, the *slipping* of the bodies in the presence of Coulomb friction leads to extended Filippov systems, and the *rolling* or *sticking* of the bodies corresponds to the sliding dynamics inside the discontinuity manifold. In the following central part of the paper, the analysis of the limit directions is applied to explore the transitions between slipping and rolling between the bodies.

## Analysis of limit directions at the rigid body

### The resulting extended Filippov system

The full phase space of the body consists of the quasi-velocities (14) and the generalized coordinates (12). Hence, the state variable vector can be written in the form

27 $$\begin{aligned} x= (s,q)=(u_1,u_2,\omega _1,\omega _2,\omega _3,q_1,\dots q_5). \end{aligned}$$

Consequently, $x\in \mathcal {D}\subset \mathbb {R}^{10}$. By composing the vector field from (18) and (15) in the form $F=(f(s,q),K(q)\cdot s)$, the dynamics of the body can be simply written as

28 $$\begin{aligned} \dot{x}=F(x). \end{aligned}$$

The vector field *F*(*x*) is discontinuous due to the terms $u_1/\sqrt{u_1^2+u_2^2}$ and $u_2/\sqrt{u_1^2+u_2^2}$ originating from the contact force (17). Equations (10)–(11) show that the final form of *F*(*x*) depends linearly on the contact force $\mathbf {F}_P$. Hence, the resulting vector field can be written in the form

29 $$\begin{aligned} F(x)=\tfrac{u_1}{\sqrt{u_1^2+u_2^2}}\cdot A(x)+\tfrac{u_2}{\sqrt{u_1^2+u_2^2}}\cdot B(x)+C(x), \end{aligned}$$

where *A*(*x*), *B*(*x*),  and *C*(*x*) are *smooth* vector fields.

The system (29) is smooth everywhere except in $u_1=u_2=0$. That is, the discontinuity set is

30 $$\begin{aligned} \Sigma =\left\{ (0,0,\omega _1,\omega _2,\omega _3,q_1,\dots q_5)\right\} , \end{aligned}$$

which is a codimension-2 (8 dimensional) discontinuity.

#### Theorem 1

The system (29) is an extended Filippov system.

#### Proof

Let us check conditions (a) and (b) in Definition 1 by calculating the limit vector field $F^*(\phi )$. As (30) is a linear subspace of the phase space $\mathcal {D}$, the corresponding orthogonal space $\mathcal {O}_{x_0}\Sigma$ is constant. Hence, independently from $x_0\in \Sigma$, we can fix the basis vectors of $\mathcal {O}_{x_0}\Sigma$ to $n_1=(1,0,\dots 0)$ and $n_2=(0,1,\dots 0)$. Then, the set of normal vectors from (21) becomes

31 $$\begin{aligned} n(\phi )=(\cos \phi ,\sin \phi , 0, \dots 0), \end{aligned}$$

and direct calculation of (23) leads to

32 $$\begin{aligned} F^*(\phi )(x_0)=\cos \phi \cdot A(x_0)+\sin \phi \cdot B(x_0)+C(x_0). \nonumber \\ \end{aligned}$$

Condition (b) in Definition 1 is satisfied because $F^*(\phi )$ exists for all $x_0$ and $\phi$. Condition (c) requires that $A(x_0)=B(x_0)=0$ cannot occur for any $x_0\in \Sigma$. These quantities come from the coefficients of the nonsmooth terms in the expression of $\mathbf {F}_P$ in (17). These coefficients are nonzero because of $\mu >0$ (there is indeed friction) and $N>0$ (required in Sect. 2.2). Therefore, the non-singular linear operations on $\mathbf {F}_P$ in (10)–(11) show that it is not possible to obtain zero for all components of $A(x_0)$ and $B(x_0)$ at the same time. Consequently, all conditions of Definition 1 are satisfied, and thus, (29) *is an extended Filippov system*. $\square$

### Analysis of the limit directions of the system

The discontinuity set $\Sigma$ of (29) is defined by $u_1=u_2=0$ (see (30)), which corresponds to the rolling of the body on the plane. In this subsection, we categorize the points of $\Sigma$ according to the number and type of *limit directions*, which are strongly connected to the transitions between rolling and slipping.

For a chosen point $x_0\in \Sigma$, the projection (24) of the limit vector field can be calculated by simply taking first two components of (32). Hence, we get

33 $$\begin{aligned} \begin{aligned} F_1^*(\phi )&=A_1\cos \phi +B_1\sin \phi +C_1, \\ F_2^*(\phi )&=A_2\cos \phi +B_2\sin \phi +C_2, \end{aligned} \end{aligned}$$

where $A_1\dots C_2$ denote the first two components of $A(x_0)$, $B(x_0)$ and $C(x_0)$ from (32) without denoting the (smooth) dependence on $x_0$. Then, the functions *R* and *V* from (26) become

34 $$\begin{aligned} R(\phi )= & {} \frac{A_1+B_2}{2}+C_1\cos \phi +C_2\sin \phi \nonumber \\&+\frac{A_1-B_2}{2}\cos 2\phi +\frac{A_2+B_1}{2}\sin 2\phi , \end{aligned}$$

35 $$\begin{aligned} V(\phi )= & {} \frac{A_2-B_1}{2}+C_2\cos \phi -C_1\sin \phi \nonumber \\&+\frac{A_2+B_1}{2}\cos 2\phi -\frac{A_1-B_2}{2}\sin 2\phi . \end{aligned}$$

From Definition 2, the limit directions are the zeroes of the function $V(\phi )$. In this section, we derive conditions from the coefficients of (33) and (34)–(35), which determine the type and number of the limit directions.

#### Possible formal simplifications

##### Proposition 1

In (33), the coefficients satisfy $B_1=A_2$.

##### Proof

Let us calculate the coefficients $A_2$ and $B_1$ formally from (10)–(11). By using the notations

36 $$\begin{aligned} \mathbf {J}^{-1}=\frac{1}{m}\cdot \begin{bmatrix} j_{11}&\quad j_{12}&\quad j_{13} \\ j_{12}&\quad j_{22}&\quad j_{23} \\ j_{13}&\quad j_{23}&\quad j_{33} \end{bmatrix} \end{aligned}$$

and

37 $$\begin{aligned} \mathbf {r}_{PC}=r_1\mathbf {t}_1+r_2\mathbf {t}_2+r_3\mathbf {n}, \end{aligned}$$

we get

38 $$\begin{aligned} \begin{aligned} A_2&=-\tfrac{\mu N}{m} (r_2r_3j_{13}+r_1r_3j_{23}-r_3^2j_{12}-r_1r_2j_{33}),\\ B_1&=-\tfrac{\mu N}{m} (r_2r_3j_{13}+r_1r_3j_{23}-r_3^2j_{12}-r_1r_2j_{33}).\\ \end{aligned} \end{aligned}$$

We obtained that $B_1=A_2$. $\square$

##### Proposition 2

The coefficients $A_1$ and $B_2$ in (38) are strictly negative for the physically relevant parameters.

##### Proof

By performing similar direct calculation as in the previous proof, we get

39 $$\begin{aligned} \begin{aligned} A_1&=-\tfrac{\mu N}{m} \left( 1+(r_2^2j_{33}-2r_2r_3j_{23}+r_3j_{22})\right) ,\\ B_2&=-\tfrac{\mu N}{m} \left( 1+(r_1^2j_{33}-2r_1r_3j_{13}+r_3j_{11})\right) .\\ \end{aligned} \end{aligned}$$

The moment of inertia tensor $\mathbf {J}$ is *positive definite*. In the inner bracket of the expression of $A_1$ in (39), the expression can be obtained as the bilinear mapping of the vector $r_3\mathbf {t}_2-r_2\mathbf {n}$ by the positive definite matrix $m\mathbf {J}^{-1}$; thus, this expression is positive. Similarly, the expression of $B_2$ contains the bilinear mapping of $r_3\mathbf {t}_1-r_1\mathbf {n}$ by $m\mathbf {J}^{-1}$, which is positive, again. If there is non-vanishing contact force ($N>0$) and there is indeed friction ($\mu >0$), then we obtain $A_1<0$ and $B_2<0$. $\square$

##### Proposition 3

Consider the transformation of the variables $u_1$ and $u_2$ defined by

40 $$\begin{aligned} \begin{aligned} u_1'&=u_1\cos \delta +u_2\sin \delta , \\ u_2'&=u_2\cos \delta -u_1\sin \delta . \end{aligned} \end{aligned}$$

Then, $\delta$ can be chosen such that the coefficients $A'_2=B'_1$ vanish, which denote the transformed coefficients corresponding to $A_2$ and $B_1$ in (33).

##### Proof

By performing the transformation (40), the relation of the original and transformed coefficients of (33) is

41 $$\begin{aligned} A'_2=B'_1=A_2\cos 2\delta +\frac{1}{2}(B_2-A_1)\sin 2\delta . \end{aligned}$$

By choosing $\tan 2\delta =2A_2/(A_1-B_2)$, the coefficients $A'_2=B'_1$ are eliminated. $\square$

It follows from Proposition 3 that with an appropriate choice of the basis vectors $\mathbf {t}_1$ and $\mathbf {t}_2$ in the tangent plane of the body, (33) can be written into the form

42 $$\begin{aligned} \begin{aligned} F_1^*(\phi )&=A_1\cos \phi +C_1, \\ F_2^*(\phi )&=B_2\sin \phi +C_2, \end{aligned} \end{aligned}$$

without the loss of generality. In this form, (43)–(44) become

43 $$\begin{aligned} R(\phi )= & {} \frac{A_1+B_2}{2}+C_1\cos \phi +C_2\sin \phi \nonumber \\&+\frac{A_1-B_2}{2}\cos 2\phi , \end{aligned}$$

44 $$\begin{aligned} V(\phi )= & {} C_2\cos \phi -C_1\sin \phi -\frac{A_1-B_2}{2}\sin 2\phi . \end{aligned}$$

#### Possible number of limit directions

In the expression of (35) and (44), $V(\phi )$ is a truncated Fourier series containing terms up to the second harmonics. According to [10], determining the zeroes of such function leads to the eigenvalue problem of a 4-by-4 complex matrix. Alternatively, finding the zeroes of $V(\phi )$ is equivalent to solving the following fourth-order polynomial equation.

##### Proposition 4

The zeroes $\phi \in [0,2\pi )$ of (44) satisfy the following equation:

45 $$\begin{aligned} ((B_2-A_1)\cos \phi -C_1)^2(1-\cos \phi )^2-C_2^2\cos ^2\phi =0 \end{aligned}$$

##### Proof

Equation (45) can be derived from (44) by direct calculation using basic trigonometric identities. $\square$

##### Proposition 5

The function $V(\phi )$ in (44) has maximum four zeroes except if $V(x_0)(\phi )$ is identically zero.

##### Proof

Equation (45) is a fourth-order polynomial in $\cos \phi$, which leads to maximum four different roots for $\phi$ on the interval $\phi \in [0,2\pi )$. In the degenerate case when $V(\phi )$ is identically zero, all $\phi \in [0,2\pi )$ are limit directions. $\square$

##### Proposition 6

The function $V(\phi )$ in (44) has minimum two zeroes.

##### Proof

In the form (44) of $V(\phi )$, the constant term vanishes. Therefore, $V(\phi )$ is a periodic continuous function with zero mean value. Thus, it needs to have at least two zeroes on $\phi \in [0,2\pi )$. $\square$

##### Proposition 7

The function $V(\phi )$ in (44) has three zeroes if and only if

46 $$\begin{aligned} C_1^{2/3}+C_2^{2/3}=(B_2-A_1)^{2/3}\ne 0. \end{aligned}$$

##### Proof

The periodic differentiable function $V(\phi )$ can have odd number of roots only if it has a double root $\phi _1\in [0,2\pi )$ with

47 $$\begin{aligned} V(\phi _1)=0,&\frac{\mathrm {d}V}{\mathrm {d}\phi } (\phi _1)=0. \end{aligned}$$

By substituting (44) into the two equations of (47), direct calculation leads to $\tan \phi _1=-(C_2/C_1)^{1/3}$ and (46). $\square$

##### Theorem 2

Consider a point $x_0\in \Sigma$ of the system (29). The following cases can occur.

1. If $C_1^{2/3}+C_2^{2/3}>(B_2-A_1)^{2/3},$ then $x_0$ has 2 limit directions.
2. If $C_1^{2/3}+C_2^{2/3}<(B_2-A_1)^{2/3},$ then $x_0$ has 4 limit directions.
3. If $C_1^{2/3}+C_2^{2/3}=(B_2-A_1)^{2/3}\ne 0,$ then $x_0$ has 3 limit directions.
4. If $C_1=C_2=B_2-A_1=0,$ then $x_0$ has continuously many limit directions.

##### Proof

Point 3 of the Theorem is contained by Proposition 7. The condition (46) separates the space of the coefficient $A_1,B_2,C_1,C_2$ into two regions, where there can be 2 or 4 limit directions (see Propositions 5 and 6). In the case $C_1^{2/3}+C_1^{2/3}\gg (B_2-A_1)^{2/3}$, the last term in (44) is negligible, that is, there are two roots of $V(\phi )$ on $[0,2\pi )$, resulting in Point 1 of the Theorem. Point 2 can be proved similarly by checking the extreme case $C_1^{2/3}+C_1^{2/3}\ll (B_2-A_1)^{2/3}$, when there are four roots of (44). In Point 4, $V(\phi )$ is identically zero and all $\phi \in [0,2\pi )$ are limit directions. $\square$

#### Attracting and repelling limit directions

##### Proposition 8

The function (44) possesses a zero satisfying $R(\phi _1)=0$ in (44) if

48 $$\begin{aligned} \frac{C_1^2}{A_1^2}+\frac{C_2^2}{B_2^2}=1. \end{aligned}$$

##### Proof

The condition $V(\phi _1)=R(\phi _1)=0$ is equivalent to $F^*_1(\phi _1)=F^*_2(\phi _1)=0$ in (42). By direct calculation, we get $\tan \phi _1=A_1C_2/(B_2C_1)$ and the condition (48) of Proposition. $\square$

##### Theorem 3

Consider a point $x_0\in \Sigma$ of the system (29). The following cases can occur.

1. If $C_1^2/A_1^2+C_2^2/B_2^2<1,$ then $x_0$ has only attracting limit directions and no repelling limit directions.
2. If $C_1^2/A_1^2+C_2^2/B_2^2>1,$ then $x_0$ has at least one attracting limit direction and exactly one repelling limit direction.
3. If $C_1^2/A_1^2+C_2^2/B_2^2=1,$ then $x_0$ has attracting limit directions and a limit directions on the boundary of being attracting and repelling.

##### Proof

In Point 3 of the Theorem, we can find the condition of Proposition 8 which ensures the existence of a limit directions between being repelling and attracting (see Definition 3). This condition (48) separates the space of the coefficient $A_1,B_2,C_1,C_2$ into two regions, and the number of the repelling (or attracting) direction changes by one when crossing the boundary (48).

In the limit case $(C_1^2+C_2^2)/\max (A_1,B_2)\rightarrow 0$, the terms with $\sin \phi$ and $\cos \phi$ are negligible in (44), and we get

49 $$\begin{aligned} R(\phi )\rightarrow \tfrac{A_1}{2}(1+\cos 2\phi )+\tfrac{B_2}{2}(1-\cos 2\phi ), \end{aligned}$$

which is always negative because $A_1,B_2<0$ (see Proposition 2). That is, there is no possibility for a repelling limit direction $\phi _1$with $R(\phi _1)>0$, and Point 1 of the Theorem is proved. Proof of Point 2 comes from the fact that exactly one limit direction changes between attracting and repelling on the curve (48). $\square$

#### The four generic cases

The boundary (46) in Proposition 7 divides the space of the coefficients $A_1,B_2,C_1,C_2$ into two typical regions (see Theorem 2). Similarly, the boundary (48) in Proposition 8 creates two typical regions (see Theorem 3). This creates four generic regions in the space of $A_1,B_2,C_1, and C_2$. For fixed values of $A_1$ and $B_2$, these regions can be visualized in the plane of $C_1$ and $C_2$.

**Fig. 4:**
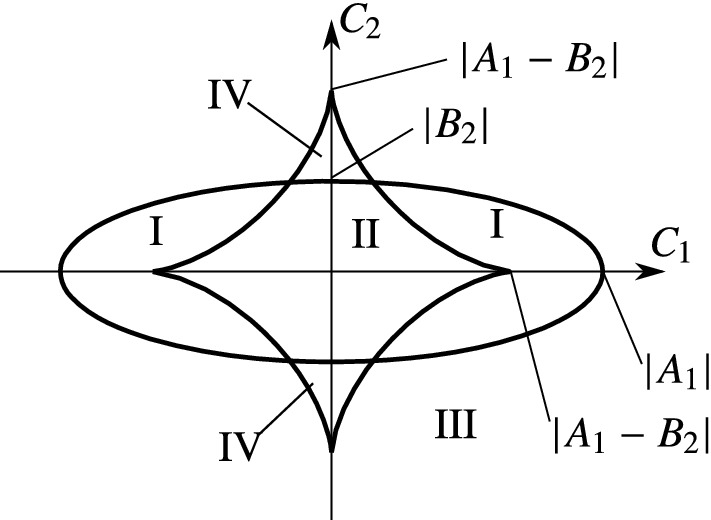
Location of the regions with different type and number of limit directions. The diagram is depicted in the space of the parameters of (33) in the case $|A_1|>|A_1-B_2|>|B_2|$. The cases I-IV are denoted in the different regions of the figure. The ellipse corresponds to (48) and the star-like boundary corresponds to (46). A similar structure of the figure is obtained for $|B_2|>|A_1-B_2|>|A_1|$

For $|A_1-B_2|>\min (|A_1|,|B_1|)$, all four types of regions appear (see Fig. 4). The boundary curves corresponding to (46) and (48). The Roman numbers denote the combinations of the cases of Theorems 2 and (3) according to the following nomenclature:

I. 2 attracting limit directions,
II. 4 attracting limit directions,
III. 1 attracting and 1 repelling limit directions,
IV. 3 attracting and 1 repelling limit directions.

**Fig. 5:**
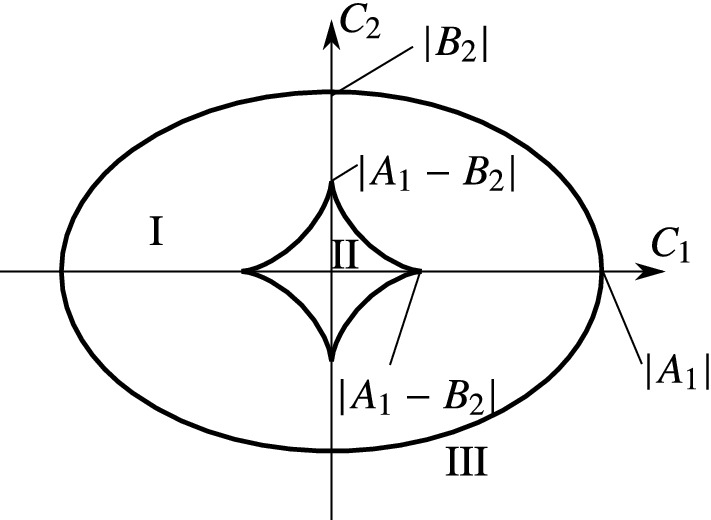
Location of the regions with different type and number of limit directions. The diagram is depicted in the space of the parameters of (33) in the case $|A_1|>|B_2|>|A_1-B_2|$. A similar structure of the figure is obtained for $|B_2|>|A_1|>|A_1-B_2|$

In the case $|A_1-B_2|\le \min (|A_1|,|B_1|)$, the case IV is absent from the plane of $C_1$ and $C_2$ (see Fig. 5). Note that the coefficients $A_1,B_2,C_1,$ and $C_2$ depend smoothly on the state variables $\omega _1\dots q_5$ from (27). Thus, the boundaries in Figs. 4–5 could be mapped onto seven-dimensional surfaces in the phase space, where they divide the eight-dimensional discontinuity set $\Sigma$ into regions according to the behaviours I-IV.

**Fig. 6:**
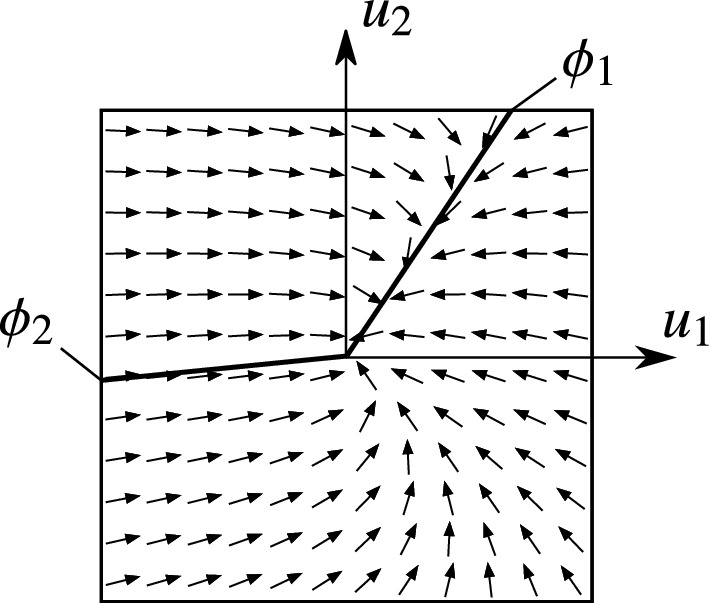
Phase portrait with the limit directions in Case I

**Fig. 7:**
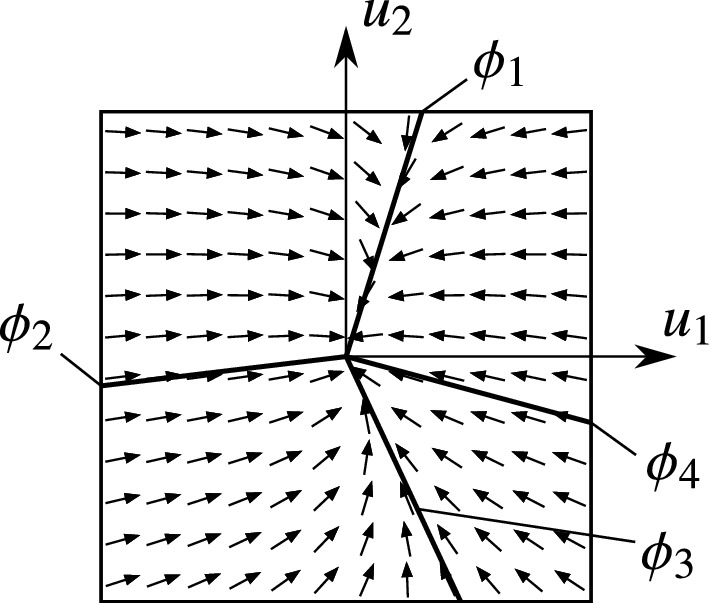
Phase portrait with the limit directions in Case II

**Fig. 8:**
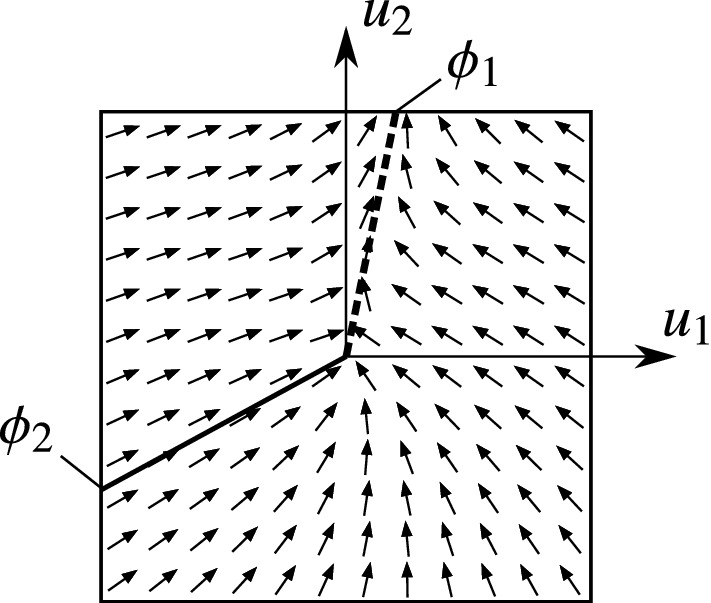
Phase portrait with the limit directions in Case III

**Fig. 9:**
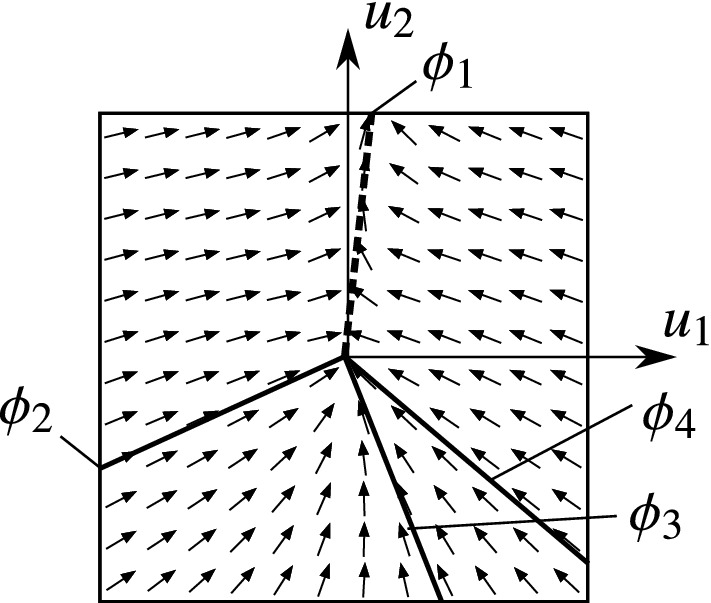
Phase portrait with the limit directions in Case IV

The typical structure of the vector field in the four cases can be seen in Figs. 6–9. The figures show the projection of the vector field into the orthogonal space $\mathcal {O}_{x_0}\Sigma$. The origin of the diagram corresponds to the given point $x_0$ of the discontinuity $u_1=u_2=0$ of the rolling behaviour. The direction of the vector field induces that the trajectories approach the discontinuity along the *attracting limit directions* (denoted by solid lines), and they leave the discontinuity along the *repelling limit directions* (denoted by dashed lines).

#### Angularly stable and unstable limit directions

We can see in Figs. 6–9 that the the trajectories are different in the neighbourhood of different attracting directions. For example in Fig. 6, most trajectories seem to be follow the direction $\phi _1$, and not $\phi _2$. This difference can be explained by the subsequent analysis.

Let us consider the system

50 $$\begin{aligned} \begin{aligned} \dot{u}_1=F^*_1(\arctan (u_2,u_1)),\\ \dot{u}_2=F^*_2(\arctan (u_2,u_1)), \end{aligned} \end{aligned}$$

which is an asymptotic approximation of projection of the system (29) into the normal plane of $u_1$ and $u_2$ in the limit case $\sqrt{u_1^2+u_2^2}\rightarrow 0$.

By using the transformation $u:=\sqrt{u_1^2+u_2^2}$, $\phi =\arctan (u_2,u_1)$, and an appropriate transformation of time, the trajectories of (50) are mapped to the trajectories of the system

51 $$\begin{aligned} u'&=u R(\phi ), \end{aligned}$$

52 $$\begin{aligned} \phi '&=V(\phi ), \end{aligned}$$

where the dash denoted the differentiation with respect to the new time variable. Note that the solutions of (52) can be determined independently from (51). The Taylor expansion of (52) around an equilibrium $\phi _1$ with $V(\phi _1)=0$ is given by

53 $$\begin{aligned} \phi '=\left. \frac{\mathrm {d}V(\phi )}{\mathrm {d}\phi }\right| _{\phi =\phi _1} \cdot (\phi -\phi _1)+\mathcal {O}^2(\phi -\phi _1), \end{aligned}$$

where $\mathcal {O}^2$ denotes the higher order terms. The linear stability of $\phi _1$ of equation (52) is determined by the sign of $\mathrm {d}V(\phi )/\mathrm {d}\phi$ at $\phi =\phi _1$. The equilibrium points of (52) corresponds to the limit directions of the original system (see Definition 2). Hence, the terms *stable* and *unstable* of the equilibrium point can be transferred to the limit directions.

##### Definition 5

The limit direction $\phi _1$ of (29) is called ***angularly stable*** if $\mathrm {d}V(\phi )/\mathrm {d}\phi$ is negative at $\phi =\phi _1,$ and it is called ***angularly unstable*** if $\mathrm {d}V(\phi )/\mathrm {d}\phi$ is positive at $\phi =\phi _1$.

In the angularly stable case, the limit direction is attracting the adjacent trajectories (see $\phi _1$ in Fig. 6). In the angularly unstable case, the adjacent trajectories get far from the limit directions in the sense of the angle $\phi$ (see $\phi _2$ in Fig. 6).

The special case $\mathrm {d}V(\phi )/\mathrm {d}\phi =0$ is a *fold bifurcation* of (52), which corresponds to the *fold* of limit directions in (29). This condition was already discussed in Proposition 7 (see (47)); thus, the fold of directions coincides with the condition (46). The next Proposition completes our analysis of the limit directions of (29).

##### Proposition 9

If a limit direction of (29) is repelling, then it is an angularly stable limit direction.

##### Proof

A limit direction can change from attracting to repelling only on the boundary (48). Let us substitute (48) and the corresponding value of $\phi _1$ from the proof of Proposition 8 into $\mathrm {d}V(\phi )/\mathrm {d}\phi$. Then, we get

54 $$\begin{aligned} \left. \frac{\mathrm {d}V(\phi )}{\mathrm {d}\phi }\right| _{\phi =\phi _1}= A_1\frac{C_2^2}{B_2^2}+B_2\frac{C_1^2}{A_1^2}, \end{aligned}$$

which is always negative due to Proposition 2. That is, the limit direction is angularly stable. $\square$

**Table 3:** Number and type of the limit directions in the four generic cases of the system

Case	I	II	III	IV
Total number of limit directions	2	4	2	4
Attracting, angularly stable	1	2	0	1
Attracting, angularly unstable	1	2	1	2
Repelling, angularly stable	0	0	1	1
Repelling, angularly unstable	0	0	0	0

To summarize our results, the number and properties of the limit trajectories can be found in Table 3.

## Slipping–rolling transitions

### Mechanical consequence of the limit directions

The system *F*(*x*) in (28) was introduced to describe the *slipping* behaviour of the body. The discontinuity manifold $\Sigma$ is the set $u_1=u_2=0$, which coincides with the condition of the *rolling* constraint (8).

In this subsection, the slipping–rolling transitions are analysed by considering purely the limit directions of the *slipping equations* determined in Sect. 4. The relation to the dynamical conditions of the *rolling equations* is presented in the next subsection.

#### Case I: 2 attracting directions

In this case, all trajectories in the vicinity of $x_0$ tend to the discontinuity at $u_1=u_2=0$ (see Fig. 6). That is, the behaviour of the body turns from slipping to rolling. It is proved in [4] that the trajectories reach $u_1=u_2=0$ in *finite* time. In some sense, the rolling motion is *stable* with respect to slipping perturbations, because the effect of a small perturbation in $u_1$ and $u_2$ is eliminated by the dynamics in finite time.

Note that *almost* all solutions reach the rolling state along the angularly stable limit direction ($\phi _1$ in Fig. 6). The angle $\phi$ can be imagined not only as an angle in the phase space but as an angle of the slipping velocity $\mathbf {v}_P$, as well. Therefore, the dominant behaviour of the limit direction $\phi _1$ causes that the slipping velocity points typically into the direction $\phi _1$ when the motion changes from slipping into rolling. There is only a single trajectory which approaches the state $u_1=u_2=0$ from the direction $\phi _2$. The trajectories close to $\phi _2$ contain a high-curvature turning when reaching $x_0$. Hence, the direction of the friction force changes rapidly just before the transition from slipping to rolling.

#### Case II: 4 attracting directions

This case has a behaviour similar to Case I: all surrounding trajectories tend to the discontinuity ($u_1=u_2=0$) in finite time (see Fig. 7. From mechanical point of view, this means that the rolling motion is realizable, because small perturbations causing slipping are eliminated by the dynamics and the body starts rolling again.

But in contrast to Case I, here, we have four limit directions and there are two angularly stable limit directions ($\phi _1$ and $\phi _3$ in Fig. 7). That is, there are two typical directions of the slipping velocity when the body is in transition from slipping to rolling. The angularly unstable directions $\phi _2$ and $\phi _4$ behave as separatrices. In the regions $\phi _4<\phi <\phi _1$ and $\phi _1<\phi <\phi _2$, all trajectories approach the rolling state along the direction $\phi _1$. In the regions $\phi _2<\phi <\phi _3$ and $\phi _3<\phi <\phi _4$, the trajectories tend to the limit direction $\phi _3$.

#### Case III: 1 repelling and 1 attracting direction

When an attracting limit direction turns into repelling, the structure of the phase changes significantly. In Case III, we can find an attracting and a repelling limit direction (see Fig. 8). The attracting direction is angularly unstable and the repelling direction is angularly stable (according to Proposition 9). That is, the typical behaviour of the system is *slipping*, and almost all trajectories avoid the discontinuity at $u_1=u_2=0$.

In the vicinity of the discontinuity set (rolling behaviour), the trajectories tend to the repelling limit direction $\phi _1$ and they diverge from the rolling state. That is, a slipping motion is generated with a typical direction $\phi _1$ of the slipping velocity. There exists one single trajectory which reaches the discontinuity, and this happens along the limit direction $\phi _2$. But the system reaches the rolling state just for a moment, and it starts slipping immediately in the direction of $\phi _2$.

#### Case IV: 1 repelling and 3 attracting directions

In Case IV, there are a repelling and three attracting limit directions, which lead to the most complicated scenario of the four cases (see Fig. 9. The two angularly unstable attracting directions $\phi _2$ and $\phi _4$ are the separatrices between two typical regions of the phase plane. Between $\phi _4<\phi <\phi _1$ and $\phi _1<\phi <\phi _2$, all trajectories avoid the discontinuity and the trajectories tend to the repelling limit direction $\phi _1$. In this sense, this case is similar to Case III, and the typical behaviour of the system is slipping.

However, in the regions $\phi _2<\phi <\phi _3$ and $\phi _3<\phi <\phi _4$, the trajectories tend to the angularly stable attracting direction $\phi _3$, and they all reach the discontinuity at $u_1=u_2=0$. There is rolling only for a moment, and the system starts slipping with a slipping velocity described by the direction $\phi _1$. In contrast to Case III, not just a single trajectory is connected to the discontinuity, but a large portion of the phase plane tends to $u_1=u_2=0$. That is, the typical long-time behaviour is slipping, but for many initial conditions, rolling can occur for a moment.

#### Summary of the typical types of behaviour

After the detailed survey of the possible types of solution, let us summarize the typical four cases of behaviour from the mechanical point of view.

##### Corollary 1

Consider a rolling state of the body and let us perturb the motion by a small amount of slipping. According to the chosen state of rolling, the typical behaviour of the body is the following:

- In Cases I and II, the perturbed body returns to rolling in finite time and then it maintains the rolling state.
- In Case I, the slipping velocity vanishes from a certain direction for almost all perturbations (*see* Fig. 6).
- In Case II, the slipping velocity vanishes from two certain directions for almost all perturbations (*see* Fig.  7).
- In Cases III and IV, the perturbed body is unable to maintain a lasting rolling state and it continues slipping.
- In Case III, the slipping velocity remains finite for almost all perturbations, thus, pure slipping continues (*see* Fig. 8).
- In Case IV, two types of behaviour occur according to the direction of the perturbation. Either, the body continues pure slipping like in Case III. Or, the slipping velocity vanishes in finite time, rolling motion occurs for a single moment, and then, the body continues slipping again (*see* Fig. 9).

### Comparison with the rolling condition of the friction law

Up to this point, we analysed the rolling–slipping transitions based on purely the phase space of the *slipping* system (29). It can be seen that a detailed, consistent structure of the behaviour can be obtained from this analysis. But what is the relation between these results and the ones from the rolling condition of the friction law with the static friction force?

The equations of the *rolling* vector field can be derived either from the Newton–Euler equations (7) with the rolling constraint (8), or directly from the limit vector field (32) of the slipping case.

In the latter case, we consider the dynamics on $\Sigma$ generated by $F^*(x)$ by a convex combination, which is called *sliding dynamics* in the terminology of Filippov systems and extended Filippov systems (see [1, 7] and [4]). In mechanical problems, we have to be careful with this terminology because sliding dynamics correspond to the mechanical *rolling* and not to the mechanical *slipping*.

At a point $x_0\in \Sigma$, we search for the *sliding vector*$F_\Sigma (x_0)\in \mathcal {T}_{x_0}\Sigma$ in the form

55 $$\begin{aligned} F_\Sigma =\int _0^{2\pi }\alpha (\phi )F^*(\phi )\mathrm {d}\phi , \end{aligned}$$

where $\alpha$ is a $[0,2\pi )\rightarrow [0,1]$ function which satisfies $\int _0^{2\pi }\alpha (\phi )\mathrm {d}\phi =1$ (a *convex* combination). By direct calculation from (32)–(33), we get that the only such sliding vector is

56 $$\begin{aligned} F_\Sigma = \frac{B_1C_2-C_1B_2}{A_1B_2-A_2B_1}A+ \frac{A_1C_2-C_1A_2}{A_1B_2-A_2B_1}B+C. \nonumber \\ \end{aligned}$$

By using the reduced form (42) in the appropriately chosen coordinates, the formula (56) simplifies to

57 $$\begin{aligned} F_\Sigma = -\frac{C_1}{A_1}A+ \frac{C_2}{B_2}B+C. \end{aligned}$$

If the body is rolling then $u_1=u_2=0$, and the dynamics of the other variables is described by the system (56). Then, the formula (9) is not valid, and the friction force $\mathbf {F}_f$ can be obtained as a *constraint force*. The *dynamic* condition of the rolling is usually expressed by the maximal admissible friction force in the form

58 $$\begin{aligned} |\mathbf {F}_f|\le \mu _0 N, \end{aligned}$$

where $\mu _0$ is the *static* friction coefficient.

When the static and dynamic friction coefficient is equal ($\mu _0=\mu$) then we can state the following theorem.

#### Theorem 4

Consider a state $x_0\in \Sigma$ of rolling, when the static friction force is known from the constraints and $\mu _0=\mu$.

1. If the rolling is strictly admitted by (58), that is, $|\mathbf {F}_f|<\mu _0 N$, then $x_0$ possesses no repelling limit directions.
2. If the rolling is not admitted by (58), that is, $|\mathbf {F}_f|>\mu _0 N$, then $x_0$ possesses a repelling limit direction.
3. In the special case $|\mathbf {F}_f|=\mu _0 N$, $x_0$ possesses attracting limit directions and a limit direction on the boundary of being attracting and repelling.

#### Proof

The *rolling dynamics* ensures $u_1=u_2=0$ permanently, that is, the derivatives $\dot{u}_1$ and $\dot{u}_2$ have to be zero, as well. If $\mu _0=\mu$ then third statement of the theorem with $|\mathbf {F}_f|=\mu _0 N$ gives back the condition (9) of the dynamic friction. That is, the rolling and slipping dynamics is valid at the same time. In the slipping dynamics, the condition $\dot{u}_1=\dot{u}_2=0$ is equivalent to the condition (48) (see the proof of Proposition 8), which decides whether there exists a repelling limit direction or not (see Theorem 3). The magnitude $|\mathbf {F}_f|$ of the friction force tends to zero when $A_1\rightarrow 0$ and $B_2\rightarrow 0$ [compare (6), (11), (17) and (29)]. Consequently, the three cases of Theorems 3 and 4 are pairwise equivalent. $\square$

**Fig. 10:**
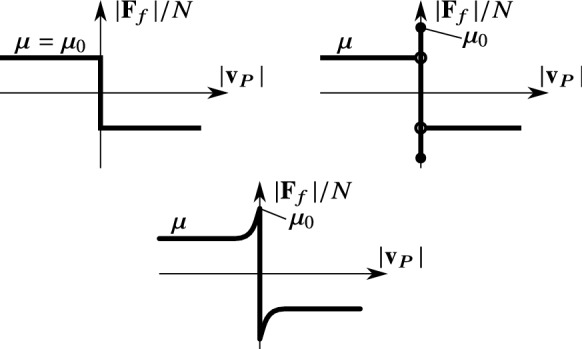
Graph of different dry friction models. Top-left: simple Coulomb model with uniform $\mu =\mu _0$ values of dynamic and static friction coefficients. Top-right: Coulomb friction with *stiction* effect, $\mu _0>\mu$. Bottom: Stribeck model without viscous effect (see (59)–(60))

The theorem is valid for the simple Coulomb model with a uniform friction coefficient $\mu _0=\mu$ (see the top-left panel of Fig. 10). However, it is not valid for the *stiction* model where there are two distinct values of $\mu$ and $\mu _0$ in (9) and (58), respectively (see the top-right panel of Fig. 10). In some sense, this stiction model provides inconsistent friction forces in the rolling and slipping cases. In this model, there are three discontinuities at $|\mathbf {v}_P|=0$: the change of the sign of the friction force and the change between the static and dynamic friction in both directions. This degeneracy can be avoided by replacing the constant dynamic coefficient $\mu$ by a function

59 $$\begin{aligned} \tilde{\mu }(|\mathbf {v}_P|)=(\mu _0-\mu )\cdot \exp (-\gamma \cdot |\mathbf {v}_P|)+\mu , \end{aligned}$$

and then, the slipping Coulomb model (9) becomes

60 $$\begin{aligned} \mathbf {F}_f=-\tilde{\mu }(|\mathbf {v}_P|)\cdot N\frac{\mathbf {v}_P}{|\mathbf {v}_P|}. \end{aligned}$$

The model (59)–(60) provides a Stribeck friction model without the viscous effect (see the bottom panel of Fig. 10). The parameter $\gamma$ can be estimated empirically, and the limit case $\gamma \rightarrow \infty$ gives back the stiction model. In fact, (59) can be replaced by any smooth function $\tilde{\mu }(|\mathbf {v}_P|)$ satisfying

61 $$\begin{aligned} \lim _{|\mathbf {v}_P|\rightarrow 0}\tilde{\mu }(|\mathbf {v}_P|)=\mu _0,&\lim _{|\mathbf {v}_P|\rightarrow \infty }\tilde{\mu }(|\mathbf {v}_P|)=\mu . \end{aligned}$$

#### Proposition 10

By considering the improved slipping friction model (60)–(61) instead of (9), Theorem 4 remains valid for different values of the static and dynamic friction coefficients.

#### Proof

By the first limit of (61), the dynamic friction coefficient tends to $\mu _0$ when $|\mathbf {v}_P|\rightarrow 0$. Then, $\mu$ can be replaced by $\mu _0$ in $F^*$ and the related quantities all along the analysis of the paper. Then, the proof of Theorem 4 can be repeated. $\square$

Theorem 4 and Proposition 10 state that the analysis of the limit directions in Sect. 4 is consistent with the checking of the maximal admissible friction force. In Cases I and II with only attracting limit directions (Figs. 6–7), the condition (58) is satisfied (rolling is realizable). In Cases III and IV with a repelling limit direction (Figs. 8–9), the condition (58) is violated (rolling is *not* realizable).

That is, Theorem 3 decides whether rolling is possible or not based on the *slipping* equations, and we do not need to calculate the rolling dynamics (56) and check the condition (58) of rolling. This property can be useful especially in those systems where it is *not possible* to calculate the static friction force (see [1, 2]). Moreover, the analysis of limit directions gives more detailed information than just deciding between rolling or slipping. The number and location of limit directions characterize the possible transitions between the slipping–rolling states and the direction of the slipping velocities at the transition.

**Fig. 11:**
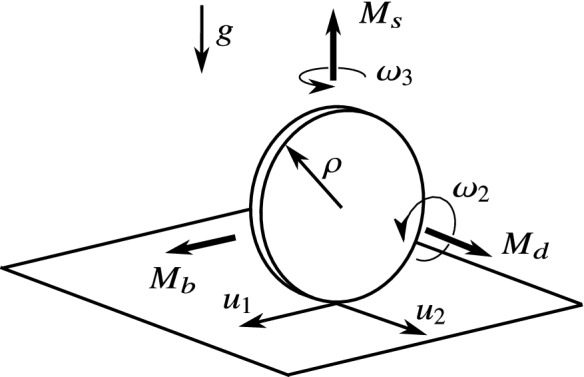
The model of the application example. The wheel is moving on the plane driven by the driving moment $M_d$ and the steering moment $M_s$. The tilting of the wheel is prevented by the balancing moment $M_b$. In this example, the occurrence of the different types of slipping–rolling transitions is shown based on the results presented in the paper

## Application example

Consider a wheel moving on a plane (see Fig. 11), where the symmetry axis of the wheel remains parallel with the plane (no tilting of the wheel). The wheel is modelled by a rigid disc with a radius $\rho$ and a negligible thickness. The external forces acting on the wheel are the gravity force (*mg*), the steering moment ($M_s$), the driving moment ($M_d$), and the balancing moment ($M_b$), ensuring the horizontal orientation of the symmetry axis. All the other notations are the same as in Sects. 2–4 (see Tables 1). It is shown in [9] that the rolling motion of this model is equivalent to the motion of the Chaplygin-sleigh, which is an important benchmark problem of nonholonomic mechanics.

It is useful to fix the basis vectors $\mathbf {t}_1$ and $\mathbf {t}_2$ to the wheel such that $\mathbf {t}_2$ is parallel to the symmetry axis of the wheel. Then, the Newton–Euler equations of the wheels in the form (10)–(11) become

62 $$\begin{aligned} \begin{aligned} \dot{\omega }_1&=0, \\ \dot{\omega }_2&=\frac{2M_d}{m\rho ^2}+\frac{2\mu g}{\rho }\frac{u_1}{\sqrt{u_1^2+u_2^2}}, \\ \dot{\omega }_3&=\frac{4M_s}{m\rho ^2}, \\ \dot{u}_1&=u_2\omega _3-\frac{2M_d}{m\rho }-3g\mu \frac{u_1}{\sqrt{u_1^2+u_2^2}},\\ \dot{u}_2&=-u_1\omega _3-\rho \omega _2\omega _3-g\mu \frac{u_2}{\sqrt{u_1^2+u_2^2}}. \end{aligned} \end{aligned}$$

The location and orientation of the wheel on the plane do not appear in (62) due to the symmetry properties of the problem. By eliminating the trivial coordinate $\omega _1\equiv 0$, as well, the state vector (27) of the system can be written into the reduced form

63 $$\begin{aligned} x=(u_1,u_2,\omega _2,\omega _3). \end{aligned}$$

Then, the vector field *F*(*x*) becomes

64 $$\begin{aligned} F(x)=\begin{bmatrix} u_2\omega _3-\frac{2M_d}{m\rho }-3g\mu \frac{u_1}{\sqrt{u_1^2+u_2^2}} \\ -u_1\omega _3-\rho \omega _2\omega _3-g\mu \frac{u_2}{\sqrt{u_1^2+u_2^2}} \\ \frac{2M_d}{m\rho ^2}+\frac{2 g\mu }{\rho }\frac{u_1}{\sqrt{u_1^2+u_2^2}} \\ \frac{4M_s}{m\rho ^2} \end{bmatrix}. \end{aligned}$$

The discontinuity set $\Sigma$ defined by $u_1=u_2=0$ is a plane of the variables $\omega _2$ and $\omega _3$, and its selected point is denoted by $x_0=(0,0,\omega _2,\omega _3)$. The limit vector field (23) becomes

65 $$\begin{aligned} F^*(x_0)(\phi )=\begin{bmatrix} -\frac{2M_d}{m\rho }-3g\mu \cos \phi \\ -\rho \omega _2\omega _3-g\mu \sin \phi \\ \frac{2M_d}{m\rho ^2}+\frac{2g\mu }{\rho }\cos \phi \\ \frac{4M_s}{m\rho ^2} \end{bmatrix}. \end{aligned}$$

In the form (32), the vectors *A*, *B*, *andC* at $x_0$ are given by

66 $$\begin{aligned} A(x_0)&=\begin{bmatrix} -3\mu g \\ 0 \\ \frac{2g\mu }{\rho } \\ 0 \end{bmatrix},\qquad B(x_0)=\begin{bmatrix} 0 \\ -g\mu \\ 0 \\ 0 \end{bmatrix},\end{aligned}$$

67 $$\begin{aligned} C(x_0)&=\begin{bmatrix} -\frac{2M_d}{m\rho } \\ -\rho \omega _2\omega _3 \\ \frac{2M_d}{m\rho ^2}\\ \frac{4M_s}{m\rho ^2} \end{bmatrix}, \end{aligned}$$

and the coefficients (33) are

68 $$\begin{aligned} \begin{aligned} A_1&=-3g\mu ,&B_2&=-g\mu , \\ B_1&=0,&A_2&=0, \\ C_1&=-2M_d/(m\rho ),&C_2&=-\rho \omega _2\omega _3. \end{aligned} \end{aligned}$$

As $A_2=B_1=0$, the system is already in the form (42), and it is not necessary to transform the variables according to (40). By substituting these into the boundary curve (48) between slipping and rolling, we get

69 $$\begin{aligned} \left( \frac{M_d}{m\rho }\right) ^2+(3\rho \omega _2\omega _3)^2=(3g\mu )^2. \end{aligned}$$

The boundary curve (46) between the 2 and 4 limit directions becomes

70 $$\begin{aligned} \left( \frac{M_d}{m\rho }\right) ^{2/3}+(\rho \omega _2\omega _3)^{2/3}=(2g\mu )^{2/3}. \end{aligned}$$

**Fig. 12:**
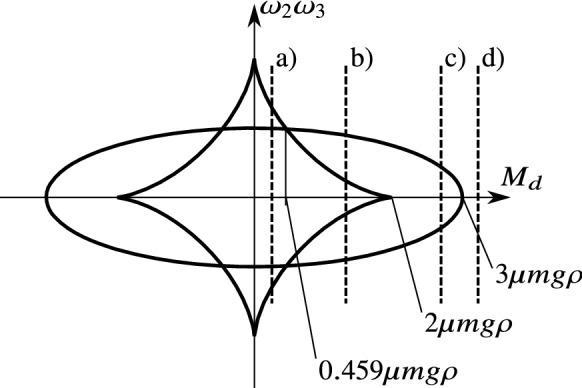
Dynamic cases at the behaviour of the rolling wheel. In the coordinate system of the driving moment $M_d$ and the product of the angular velocities $\omega _2$ and $\omega _3$, the curves separate the different regions of behaviour from the point of view of slipping and rolling. For some typical values of the parameter $M_d$, the sketch of the plane $\omega _2-\omega _3$ can be found in Fig. 13

**Fig. 13:**
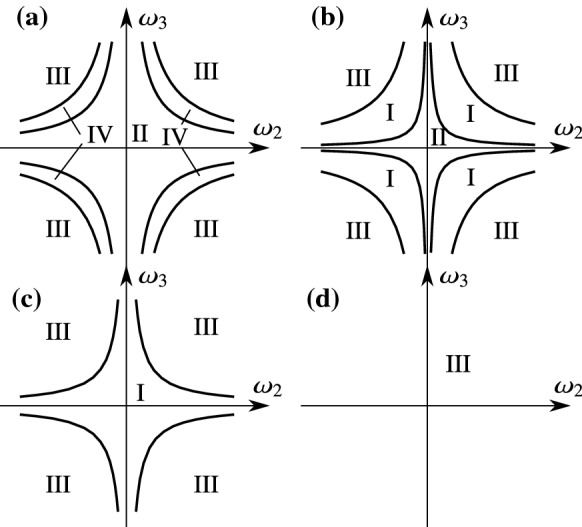
Sketch of the discontinuity set of the rolling wheel with the typical regions of behaviour. The discontinuity set is the plane of the variables $\omega _2$ and $\omega _3$. For the selected values of the driving moment $M_d$ (denoted by a–d) in Fig. 12), the different regions correspond to the four cases I–IV of behaviour presented above (see Figs. 6–9)

These curves are visualized in Fig. 12 in a similar diagram similar to Fig. 4. There are three special values of $|M_d|$, which is the absolute value of the driving moment: $|M_d|=3\mu m g\rho$, $|M_d|=2\mu m g\rho ,$ and $|M_d|\approx 0.459\mu m g\rho$ (see Fig. 12). By selecting typical values of $M_d$ in between these special values, the sketch of the discontinuity set can be found in Fig. 13. The discontinuity set $\Sigma$ is the plane of the variables $\omega _2$ and $\omega _3$. In this plane, the boundary curves are transformed into hyperbolas due to the product $\omega _2\omega _3$ in the expressions. It is not surprising that the rolling is realizable (Cases I and II) when the product of the angular velocities are not larger than a critical value. Note that the 4 limit directions (Cases II and IV) appear at low values at the angular velocities.

## Overview of more complex contact models

The results of the paper are based on the following modelling assumptions of the contact between the bodies:

1. the assumption of *planar geometry* of the fixed body,
2. the assumption of *rigid bodies*,
3. assuming that the contact state is *either slipping or rolling*,
4. the assumption of the *Coulomb friction model*,
5. the assumption of a well-defined, *single contact point* of the unloaded bodies.

In this section, we overview the possibilities of the extension of the analysis in the case of any of these restrictions are released.

If we replace the fixed plane by a rigid body with an arbitrary curved fixed surface, then the normal plane of the contacting surfaces is changing during the motion. It would make the dynamic equations more complicated, but it would not be a structural modification of the model. *Thus, the different scenarios of the limit directions are expected to be preserved in this case.*

If we do *not* neglect the deformation of the bodies, then we have to consider the formation of the *contact area* around the theoretical contact point. In the literature, it is usual to separate the motion into two parts: the rigid body motion of the whole body and the *local deformations* in the vicinity of the contact area. The Hertz theory (see, e.g. [25], p. 55 or [19] p. 84) assumes elliptical contact area and a parabolic distribution of the normal pressure between the bodies in the *frictionless* case. Similar but higher order theories exist for the normal pressures (see, e.g. [20]). Combining these models with *friction* leads to theoretical and computational challenges [19, 20, 25]. For the purpose of dynamical applications, analytical and semi-analytical models of the contact forces can be derived from these theories. When we consider the combined effect of the slipping and drilling motion, the contact laws are determined by the *Coulomb-Contensou friction model* (see, e.g. [21]). The combined effect of slipping and rolling motion leads to the contact laws of *creep models* (see, e.g. [18]). By improving our analysis by these models, *higher-codimensional (3-5) discontinuities are expected to appear*. This can be the topic of the further research work. The concept of limit directions probably remain important in these cases, as well, to find the possible transitions between rolling and slipping.

We assumed that the normal contact force *N* is strictly *positive*, and the surfaces remain in permanent contact at the contact point. Then, depending on the state of the system, the friction model decides whether slipping or rolling behaviour occurs. However, when the contact force *N* decreases to zero, the bodies can separate from each other (*lift-off*), and the *nonsmooth behaviour of the dynamics with the discontinuity set vanishes*. However, the switching of the contact and the no-contact states introduces a further discontinuity, containing the impacts of the bodies, as well. The generalization of the results of the paper to these cases would need additional extensive research work. The Painlevé paradox of the contact states [11, 15] causes further complications.

In the analysis, we first considered the simple Coulomb law for modelling dry friction. Then, in Proposition 10, the results are generalized for a class of friction models similar to the Stribeck friction law. However, several different friction models can be found in the literature (see [22] and [23] for an overview). It is an open question how further effects like hysteresis (e.g. the Karnopp model) or internal variables (e.g. the Dahl model) modify the qualitative structure of the dynamics at the discontinuity.

A further complication can be the coexistence of *multiple* contact points between the contacting bodies. The results in [1] show that the concept of limit directions is applicable to two contact points, but still, a throughout analysis would be necessary. An even further case is the contact of *conforming bodies*, where there is a finite contact area even with the rigid body assumption. (The simplest example is a block moving on a plane.) Then, information is needed about the normal pressure distribution, and the integration on the contact area is expected to lead to higher-codimensional discontinuities as we expected in the deformable models, too.

## Conclusion

The dynamical equations of a rigid body were derived, in which body is in 3D slipping or rolling contact with a rigid plane in the presence of dry friction. It was shown that by assuming Coulomb friction model, the differential equations of the dynamics of the body lead to an extended Filippov system. That is, the phase space of the system contains a codimension-2 discontinuity set.

The nonsmooth differential equations of slipping were analysed by the methods of new theory of extended Filippov systems. The possible number and type of limit directions of the points of the discontinuity were determined, where the transitions between slipping and rolling occur. We got four structurally different cases of limit directions; there can be two or four limit directions, from which maximum one can be repelling. The effect of these scenarios of the mechanical behaviour was discussed in detail. It was shown that in case of simple Coulomb model and the Stribeck model, the limit directions lead to such conditions of rolling, which are consistent with the condition of maximum admissible friction force. Furthermore, the results of the new approach provides more information about the qualitative behaviour of these mechanical systems near the discontinuity. The result was demonstrated on an example of a wheel. A part of the further work would be to apply the results to other well-known systems such as the rolling disc (see [5, 8]) and the classical skate problem (see, e.g. [9]).

The considered contact model is clearly the simplest mechanical model which is capable to describe the problem of the different possible directions of transitions between rolling and slipping in three dimensions. However, several possibilities were presented in the last section to improve the contact model in different ways.

One further important direction of the subsequent research would be to utilize these results to develop effective and reliable numerical methods for simulation of these systems. The information of the structure of the trajectories and limit directions could help to find appropriate event-driven strategies similar to those of classical Filippov systems [24].
